# The Role of Chloride Channels in Plant Responses to NaCl

**DOI:** 10.3390/ijms25010019

**Published:** 2023-12-19

**Authors:** Lulu Liu, Xiaofei Li, Chao Wang, Yuxin Ni, Xunyan Liu

**Affiliations:** 1 College of Life Sciences and Oceanography, Shenzhen University, Shenzhen 518060, China; liululu124@hotmail.com; 2 College of Physics and Optoelectronic Engineering, Shenzhen University, Shenzhen 518060, China; 3 College of Life and Environmental Sciences, Hangzhou Normal University, Hangzhou 311121, China; lxf17633576763@163.com (X.L.); wang612974@163.com (C.W.); a2274855027@163.com (Y.N.)

**Keywords:** chloride channel, plants, NaCl, Cl^−^ stress, biological functions

## Abstract

Chloride (Cl^−^) is considered a crucial nutrient for plant growth, but it can be a challenge under saline conditions. Excessive accumulation of Cl^−^ in leaves can cause toxicity. Chloride channels (CLCs) are expressed in the inner membranes of plant cells and function as essential Cl^−^ exchangers or channels. In response to salt stress in plants, CLCs play a crucial role, and CLC proteins assist in maintaining the intracellular Cl^−^ homeostasis by sequestering Cl^−^ into vacuoles. Sodium chloride (NaCl) is the primary substance responsible for causing salt-induced phytotoxicity. However, research on plant responses to Cl^−^ stress is comparatively rare, in contrast to that emphasizing Na^+^. This review provides a comprehensive overview of the plant response and tolerance to Cl^−^ stress, specifically focusing on comparative analysis of CLC protein structures in different species. Additionally, to further gain insights into the underlying mechanisms, the study summarizes the identified CLC genes that respond to salt stress. This review provides a comprehensive overview of the response of CLCs in terrestrial plants to salt stress and their biological functions, aiming to gain further insights into the mechanisms underlying the response of CLCs in plants to salt stress.

## 1. Introduction

The primary substance that causes salt damage in saline soils is NaCl [1]. In many regions of the world, the adverse effects of salinity on plants pose a significant challenge to agriculture and environmental sustainability. Plants adapt to salt stress by maintaining an intracellular osmotic balance and ion homeostasis, reducing shoot sodium (Na^+^) and chloride (Cl^−^) accumulation, and changing the architecture of the plant root to suppress ion absorption or expel ions from cells [2,3,4]. Current research focuses on salt stress induced by elevated levels of Na^+^. This encompasses studies into linked signaling pathways, including Na^+^ transport, intracellular regulation, and molecular regulatory mechanisms [5]. Chloride is not easily absorbed by soil particles because it does not form complexes and remains free in water. As a result, it is readily absorbed by plant roots, leading to widespread Cl^−^ toxicity. Climate change and human activities also contribute to the excessive Cl^−^ levels, which far exceed the amount needed for normal plant growth [6].

Several studies have shown that the transport and excretion of Cl^−^ in plant shoots are correlated with plant salt tolerance of species, especially in legumes such as *Trifolium*, *Medicago sativa*, *Glycine max*, and *Lotus corniculatus* [7,8], and in woody plants such as *Pinus tabulaeformis*, *Citrus reticulata*, and *Vitis vinifera* [9,10]. Experiments have demonstrated that Cl^−^ rather than Na^+^ is responsible for leaf damage in *Citrus reticulata Blanco*, which reduces the fruit growth and yield. Although the adverse effects of salinity on fruit yield and quality are well known, the study showed that a sulfate treatment had a comparatively lesser effect than a chloride treatment, especially at moderate conductivity levels. This indicates that Cl^−^ has a more pronounced effect on plants compared to SO_4_^2−^ [11]. Therefore, it is of relevance to delve into the response of plants to Cl^−^ stress under salt stress, the accumulation pathways, and the relationship of Cl^−^ channel genes with salt tolerance. In recent years, researchers have re-examined the role of Cl^−^ in plant salt tolerance. The investigation of Cl^−^ transport pathways and physiological effects in response to salt stress in plants has received increasing attention. In this paper, we review Cl^−^ absorption and transport in plants, the types of Cl^−^ channels in plants, and the response and tolerance of plants to Cl^−^ stress under salt stress conditions.

## 2. Chlorine Toxicity and Its Impact on Plant Metabolism

Chloride is an essential micronutrient in higher plants. It occurs primarily in the form of inorganic chloride salts. It is selectively transported by secondary activated chloride channels and transporters. Cl^−^ is transported mainly by the symplastic pathway [12]. It is involved in various processes such as intra- and extracellular osmoregulation, the maintenance of turgor pressure, and the maintenance of the stability of the membrane potential and electrical excitability [6]. In addition, Cl^−^ can indirectly affect plant photosynthesis and growth by regulating stomatal aperture and the activity of specific enzymes in plants [13,14]. It also functions as a component of certain plant hormones [15]. Notably, excessive accumulation of both Na^+^ and Cl^−^ is detrimental to plants, with studies indicating that Cl^−^ accumulation can be more detrimental than Na^+^ in certain species such as wheat, barley, citrus, grape, and avocado [15,16,17,18]. Consequently, an increasing number of biologists have turned their attention to elucidating the molecular mechanisms of Cl^−^ homeostasis in plants.

Chlorine tolerance varies among crops. Plant metabolism can be adversely affected when the critical threshold is exceeded, resulting in impaired chlorine metabolism in the plant. The harmful effects of chlorine are primarily caused by the disintegration of cellular ultrastructures such as the mitochondrial matrix and cristae structures. This concentrates the mitochondrial matrix and unevenly textures the cell wall matrix. As a result, plasmolysis occurs, the cell membrane system is destroyed, and the growth and development of plants are inhibited [11]. Excess Cl^−^ can disrupt the plant’s potential balance by inducing changes in the composition and structure of the plasma membrane, thereby increasing its permeability. As a consequence, plants may exhibit toxic symptoms, such as stunted growth, dwarfed morphology, chlorosis, and premature leaf abscission. However, in Cl^−^-tolerant desert plants, such as *Pugionium cornutum*, the use of Cl^−^ to enhance growth under salt and drought stress has been observed. This species can effectively absorb and accumulate significant amounts of Cl^−^ in saline–alkaline soils, facilitating osmotic adjustment and turgor generation. These ions penetrate the roots through anion channels and contribute to the maintenance of nitrate ion (NO_3_^−^) homeostasis in the shoots. As the most abundant anion in higher plants, Cl^−^ remains poorly studied at physiological and molecular levels despite its importance for plant nutrition and osmoregulation. Therefore, a comprehensive investigation of the mechanisms by which Cl^−^ affects plant growth and development is imperative [19].

## 3. Species and Biological Functions of CLCs

### 3.1. Diversity of CLC Protein Structures among Species

In 1990, CLC-0 was first cloned from *Torpedo marmorata*, marking the first CLC cloned. This subsequent research identified multiple coding genes in this family, which are found in animals, microorganisms, and fungi, these genes encode either Cl^−^ channels in the plasma membrane or vesicle Cl^−^/H^+^ exchangers [20]. In 2002, researchers determined the three-dimensional crystal structure of the CLC proteins present in *Salmonella typhimurium* and *E. coli murine*. The structure showed two distinct aqueous pores that were identical and independent, formed by the individual subunits of a homologous dimer membrane protein [21]. Subsequently, researchers resolved a number of CLC protein structures, including those from red algae [22], humans [23], and *Arabidopsis thaliana* [24]. CLC proteins act as exchangers in bacteria and red algae, and plants are known as exchangers, while, in mammals, hCLC-1 serves as an ion channel. Despite their highly similar structures, they exhibit different characteristics in ion conduction due to variations in the residues involved in forming the ion-conducting pore.

In various species, CLC proteins exhibit both structural and functional diversity. These proteins are defined by an ion-conducting transmembrane structure comprising 16–18 α-helices, followed by a C-terminal cystathionine ß-synthase (CBS) domain. Existing as homodimers, each subunit forms its own ion channel, which influences ion selectivity and function through conserved residues such as gating glutamate, proton glutamate, and selectivity filter serine/proline [21,22,23,24]. Exchangers and channels typically belong to different structural families, but CLC proteins prove to be an exception. CLCs have a gated pore and act as passive Cl^−^ conduits, enabling ions to diffuse along their electrochemical gradients. In contrast, transporters (CLC exchangers) display a pore that has two gates, which never open simultaneously [25]. CLC exchangers, however, facilitate the exchange of two Cl^−^ ions and one H^+^ ion in opposite directions, which calls for energy input from an external source [26]. 

**Figure 1:**
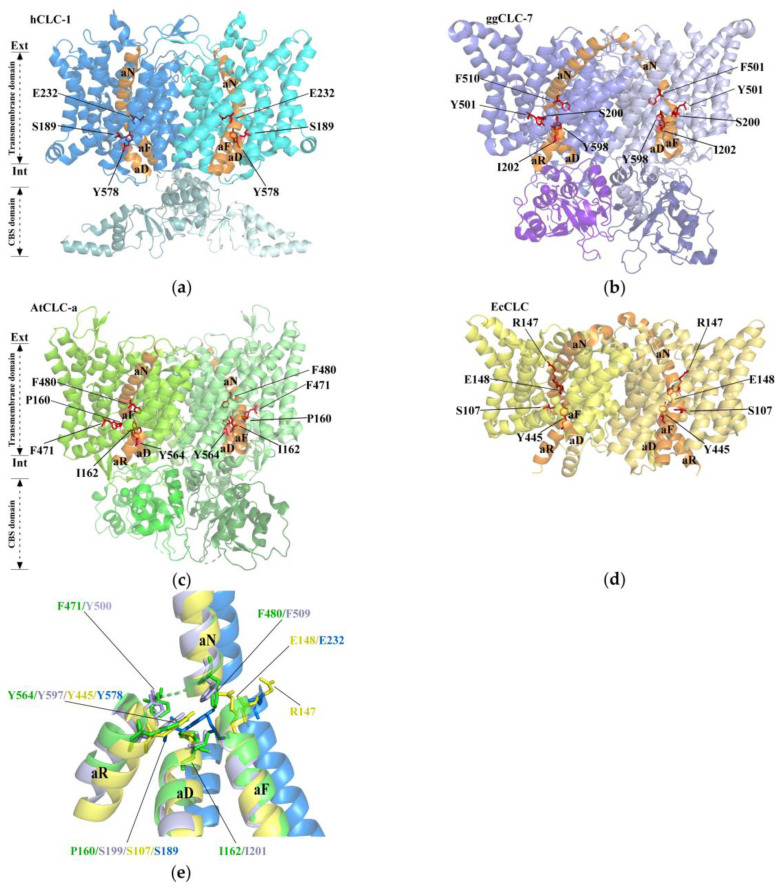
Cryo-EM structure of the CLCs. (**a**) Human CLC-1 channel hCLC-1 (PDB: 6COY and 6COZ), the transmembrane domain (TMD, blue), and the cytosolic domain (CTD, light blue). (**b**) Chicken CLC chloride–proton exchanger ggCLC-7 (PDB: 7JM6); TMD, purple; CTD, light purple. (**c**) *Arabidopsis thaliana* CLC transporter AtCLCa (PDB: 8IAB); TMD, green; CTD, light green. (**d**) *Escherichia coli* CLC channel EcCLC (PDB: 1OTS); the two subunits are yellow. The selectivity filter residues of CLCs are shown with red. (**e**) The anion selectivity filter of hCLC-1 (blue), AtCLCa (green), ggCLC-7 (purple), and EcCLC (yellow) were compared between structures.

In the Cryo-EM structure of CLCs, Cl^−^ transport is closely associated with three unique sites: S_ext_, S_cen_, and S_int_ [27]. It functions as a channel or exchanger, both have a funnel that houses a channel, running from the extracellular to the intracellular side. The crucial disparity arises from the Glugate side chains’ occupancy in the transporter, occupying S_ext_ and S_cen_ and thus prohibiting the creation of a continuous pore [22,24]. Conversely, in the channel, S_ext_ remains unoccupied, which enables the identification of a continuous pore [23]. Cl^−^ with a radius of approximately 1.7 Å is able to permeate the chloride channel by passing through an anion-selective filter comprising specific helices and residues (Figure 1). E148 and E203 are two important amino acid residues of the EcCLC [21]. E148, known as the “gate glutamate”, is located near the selectivity filter and participates in the selective transport and gating of Cl^−^. E203, also known as the “proton glutamate”, is situated on the cytoplasmic side, enabling proton transport [25,26]. If these two residues mutate, the double mutation E148A/E203Q in the EcCLC protein completely abolishes proton transport and pH sensitivity of Cl^−^ transport. The resulting double mutant exhibits maximum Cl^−^ flux under different pH conditions but does not perform any proton pumping activity [26].

Compared to that on the Cl^−^ channel protein found in animals, research on plant CLC proteins remains relatively underdeveloped. Nevertheless, recent breakthroughs, including the crystal structure analysis of AtCLC-a, have yielded crucial insights. The anion-selective filter in the plant transporter AtCLC-a has a crucial residue, Pro160, which is necessary for NO_3_^−^ selectivity in plant CLCs and replaces SerC present in animal CLCs. At the center of AtCLC-a, there is a 1.4 Å shift of NO_3_^−^ relative to Cl^−^ when compared to CLC-7 (Figure 1e). In addition, the side chains of Phe471, Phe480, and Tyr564 surrounding the central binding site exhibit noticeable outward shifts concerning anions (Figure 1c). These structural rearrangements seem to accommodate the larger NO_3_^−^, explaining the preference of AtCLC-a for NO_3_^−^. Additionally, the CBS domain is thought to serve a regulatory purpose by possibly interacting with ligands or signaling molecules, thereby influencing the activity of the CLC protein [22,24]. This study provides valuable insights into the functional mechanisms of plant CLC proteins, paving the way for further investigation.

### 3.2. Biological Functions of CLCs in Plants

#### 3.2.1. CLC Family in *Arabidopsis thaliana*

The *Arabidopsis thaliana* CLC family consists of seven members, namely, AtCLC-a/b/c/d/e/f/g, which include the NO_3_^−^/H^+^ exchanger and Cl^−^/H^+^ exchanger. Among these, AtCLC-a/b/d act as NO_3_^−^ transporters. In 2006, a patch-clamp technique was utilized to unveil the transport activities of plant CLCs in membrane systems. AtCLC-a is recognized as an NO_3_^−^/H^+^ exchange protein, which can specifically amass nitrates in a vacuole. The antiporter mechanism of CLC protein is directly correlated with its physiological role [28]. *AtCLC-b* and *AtCLC-a* share a 72% identical cDNA sequence and are recognized as vacuolar NO_3_^−^/H^+^ exchangers [29,30]. Knockout experiments of *clc-a* and *clc-b* demonstrated that only the former leads to a reduction in the nitrate fraction in *Arabidopsis* [28]. Patch-clamp experiments have demonstrated that *CLC-b* facilitates the release of anions from the vacuole, rather than the process of anion entry into the vacuole. Although the current elicited by *CLC-b* closely resembles that of *CLC-a* in oocytes, marked differences in anion selectivity and time-dependent activation exist [29]. In 2004, Harada localized the *AtCLC-c* gene by quantitative trait loci (QTL) analysis and confirmed its role in regulating nitrate levels in *Arabidopsis* [31]. Jossier reported that *AtCLC-c* is associated with stomatal movement and salt tolerance [13]. It is located on the tonoplast, and its expression is significantly upregulated in guard cells and even entire plants after ABA and salt stress treatments. Notably, *clc-c* mutants display reduced levels of nitrate, as well as altered concentrations of Cl^−^, malate, and citrate [31,32]. Similarly, Nguyen found that AtCLC-g, located on the tonoplast, was extensively expressed in mesophyll cells and showed high concordance (62%) with AtCLC-c, this correlation was associated with salt tolerance [33]. *AtCLC-g*, like *AtCLC-c*, plays a critical role in excess Cl^−^ tolerance and is an essential component of the regulatory network for Cl^−^ sensitivity. Intracellular nitrate accumulation relies on the activity of proton pumps and CLC proteins in the tonoplast [34]. *AtCLC-e* regulates Cl^−^ homeostasis during light-to-dark transitions, thus affecting chloroplast ultrastructure and photosynthetic electron movement [35].

The CLC protein has been discovered in different organelles of plant cells, such as the vacuole, Golgi, and chloroplasts. In *Arabidopsis thaliana*, the AtCLC-a, AtCLC-c, and AtCLC-g proteins are located on the tonoplast, while AtCLC-f is localized on the membrane of the Golgi vesicle [36]. AtCLC-d and VHA-a1, both proton-transporting V-type ATPases, exist within the trans-Golgi tubular reticulum. The insertion of T-DNA into the *AtCLC-d* gene did not affect the levels of nitrate and Cl^−^. Mutant *clc-d-1* plants have similar morphology to that of the wild type, however, they showcase impaired root growth when placed in a medium and exhibit a higher sensitivity to concanamycin A, a blocker of V-ATPase. Overexpression of AtCLC-d in the mutant complements the phenotype, indicating a role for AtCLC-d in the regulation of the trans-Golgi cavity pH through Cl^−^ or NO_3_^−^ transport [37]. AtCLCe governs chloroplast Cl^−^ balance during light–dark transitions, influencing thylakoid ultrastructure and photosynthetic electron transport regulation (Table 1) [35]. When examining the connection between Cl^−^ and salt tolerance, it is important to also take into account the presence of NO_3_^−^. *Arabidopsis* CLCs (AtCLC-a and AtCLC-b) exhibit greater selectivity for NO_3_^−^ compared to Cl^−^, and NO_3_^−^/Cl^−^ interactions are akin to K^+^/Na^+^ interactions during the Na^+^ exclusion mechanism when subjected to salt stress. In terms of maintaining charge equilibrium, Cl^−^ uptake can either be balanced by uptake of another cation such as Na^+^ or by the loss of another anion such as NO_3_^−^ [18].

#### 3.2.2. Functional Studies of CLC Proteins in Various Species of Terrestrial Plants

**Figure 2:**
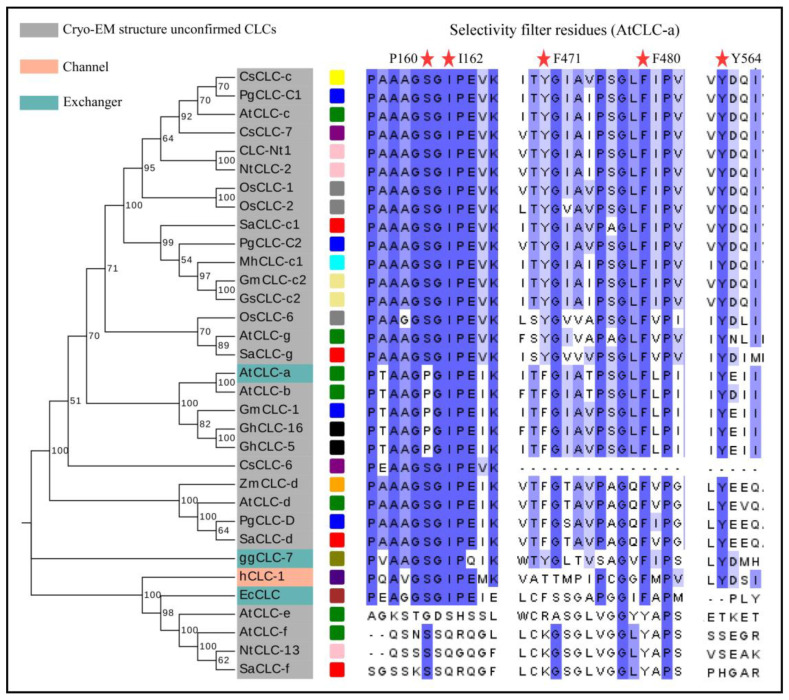
Phylogenetic analysis and conserved motif analysis of CLC proteins. The full-length amino acid sequences of CLC proteins were aligned by Clustal W, and the phylogenetic tree was built using the neighbor-joining (NJ) method in MEGA (v11.0). The grouping of the CLC proteins is indicated by different colors. AtCLC-x (green box); CsCLC-c (yellow box); CsCLC-x (purple box); EcCLC (brown box); ggCLC-7 (olive box); GmCLC-x (khaki box); GsCLC-x (khaki box); GhCLC-x (black star); hCLC-1 (indigo box); MhCLC-x (cyan box); NtCLC-x (pink box); OsCLC-x (gray box); PgCLC-x (blue box); SaCLC-x (red box); ZmCLC-x (orange box). The red star represents the positions of residues.

Various CLCs have been identified in multiple plant species, including *Arabidopsis thaliana* (AtCLC-x), *Camellia_Sinensis* (CsCLC-x), *Citrus sinensis* (CsCLC-x), *Glycine max* (GmCLC-x), *Glycine soja* (GsCLC-x), *Gossypium hirsutum* (GhCLC-x), *Nicotiana tabacum* (NtCLC-x), *Oryza sativa* (OsCLC-x), *Poncirus trifoliata* (PtrCLC-x), *Punica granatum* (PgCLC-x), *Solidago altissima* (SaCLC-x), and *Zea mays* (ZmCLC-x) [28,40,41,42]. The transmembrane domain (TMD) of CLC proteins is highly conserved across various species. Nonetheless, there exist disparities in the anion-selective filter and the C-terminal CBS domains, which affect their selectivity and regulatory mechanisms for Cl^−^ or NO_3_^−^. In plants, CLC proteins are divided into two lineages, AtCLC-e and AtCLC-f belong to a lineage, and they are more similar to prokaryotic CLC proteins. Meanwhile, the other CLCs of *Arabidopsis* are in a separate lineage, suggesting that they may have originated from two distinct ancestral genes (Figure 2).

The tobacco *CLC-Nt1* gene encodes a 780-amino-acid protein with several putative transmembrane regions. The team noted that CLC-Nt1 has a 24–32% homology with CLC-0, which is part of the chloride channel family in animals. This suggests the existence of the CLC gene family in plants. Rice *OsCLC-1* and *OsCLC-2* appear to function as antiporters, exhibiting gate control and proton glutamate characteristics similar to *AtCLC-c* and *AtCLC-d* [30]. According to Nakamura et al., the homologous genes *OsCLC-1* and *OsCLC-2* have an 87.9% homology to tobacco *CLC-Nt1*. These proteins are located on tonoplast and play a crucial role in facilitating ion transport across the vacuole [41]. GmCLC-1 is a protein discovered in soybeans, it is located on the vesicle membrane, which separates ions into the vesicles to alleviate their harmful effects in the cytoplasm. Additionally, it enhances salt tolerance in transgenic plants. Overexpressing *GmCLC-1* could improve salt tolerance in poplars by reducing membrane structural damage, improving osmoregulation, and regulating antioxidant enzymes during salt stress [40,43]. Similarly, *GsCLC-c2* assisted in regulating anion homeostasis in both transgenic *Arabidopsis* and soybean plants under salt stress (NaCl), resulting in a lower Cl^−^/NO_3_^−^ ratio and improved Cl^−^/salt resistance. As mentioned above, Pro160 serves as the residue responsible for NO_3_^−^ selectivity in AtCLC-a. The identity of P160 residue in GmCLC-1 strongly suggests its likely role as an NO_3_^−^/H^+^ exchanger. However, the residue of GsCLC-c2 at this site is “S”, suggesting that it may be an Cl^−^/H^+^ exchanger (Figure 2) [44].

It was found that the chloride channel gene CsCLC-c in *Poncirus trifoliate* played a vital role in salt tolerance. Overexpression of *CsCLC-c* in *Arabidopsis* led to a significant reduction in Cl^−^ content in roots and stems, resulting in increased salt tolerance compared to that of *clc-c* mutants and the wild type [17]. *Arabidopsis AtCLC-d* is a PAMP-induced negative regulator of immunity and regulated by the FLS2 complex [39]. The maize chloride channel gene *ZmCLC-d* also plays a crucial role in improving plant stress resistance [45]. Plants overexpressing *ZmCLC-d* showed a higher expression of stress-related genes including *C-repeat binding factor* (*CBF*) *1*, *CBF2*, *CBF3*, *dehydration-responsive element-binding factor (DREB)2A*, and *rare cold*-*inducible* (*RCI) 2A* (Table 2).

The increased expressions of *SaCLC-d*, *SaCLC-f*, and *SaCLC-g* in the leaves of the halophytes *Suaeda glauca*, accompanied by the clumping of Cl^−^ within the leaf cells when subjected to different concentrations of salt stress, suggest a linear relationship with the salt stress concentration. Therefore, these proteins may have a role in segregating Cl^−^ in organelles [58]. In pomegranate leaves, *PgCLC-c1*, *PgCLC-c2*, and *PgCLC-d* showed high expression levels and were likely responsible for Cl^−^ sequestration into vacuoles [56]. *GhCLC-5/16* regulated Cl^−^ and NO_3_^−^ transport, interactions, and homeostasis, improving the Cl^−^/salt resistance in cotton [49]. *CsCLC-*c was observed to subfunctionalize in response to Cl^−^ and fluoride ions (F^−^). It has been suggested that *CsCLC–6* and *CsCLC–7* may participate in Cl^−^ uptake and long-distance transportation [47]. Conversely, *AtCLC-d* negatively regulates pathogen-associated molecular pattern-triggered immunity (PTI) [39]. NtCLC-1 in tobacco was found to co-localize and interact with the potato virus Y (PVY) 6K2 protein. This interaction caused the endoplasmic reticulum to become more alkaline, which is essential for the virus to replicate within cells and infect the entire system [60]. In summary, plant CLCs are crucial for transporting and storing Cl^−^, NO_3_^−^ transport, resisting abiotic stress resistance, and responding to plant defense.

We present a thorough analysis of established plant CLC gene sequences and their corresponding responses to NaCl, along with a phylogenetic analysis that delineates distinct clades of the CLC gene tree, with the majority of plant CLCs forming a singular class. AtCLC-e, AtCLC-f, NtCLC-13, and SaCLC-f exhibit greater affinities to bacteria (Figure 2). The structural diagram of AtCLC-a reveals the presence of five significant amino acids within the anion selectivity filter. These are P160, I162, F471, F480, and Y564. We have also listed the specific regions where these five amino acid residues are situated. It is observed that the CLC gene family members in plant clade exhibit highly conserved regions that are associated with anion selectivity. Within the first conserved region, if the residue x is serine (S), preferential transport of Cl^−^ takes place, whereas substitution with proline (P) facilitates the transport of NO_3_^−^ [60]. Hence, GhCLC-5/16 may function as an NO_3_^−^/H^+^ exchanger. In contrast to proteins from the plant clade, AtCLC-e, AtCLC-f, NtCLC-13, and SaCLC-f exhibit significant distinctions for the remaining four crucial residues, in addition to P160 (Figure 2).

## 4. The Role of CLC Proteins in Plant Response to NaCl Stress

Chloride can enter root cells through active and passive influx mechanisms. Meanwhile, the outflow of Cl^−^ from the epidermal cells of roots is dependent on electrochemical gradients [15,61]. Nitrate Transporter 1/Peptide Transporter (NPF)2.4 protein contributes to the loading of Cl^−^ in the xylem of *Arabidopsis*. AtNPF2.4 and AtNPF2.5, which belong to the nitrate excretion transporter (NAXT) subfamily, take part in the transportation in the root cortical cells [61]. Additionally, the transportation function for nitrates is widely regarded as a distinguishing feature of the NPF family of plants. Within this family, AtNPF6.3, also recognized as AtNRT1.1, is the first plant member recognized as a nitrate transporter in *Arabidopsis*. It is noteworthy that NPF7.3 (NRT1.5) serves as a transporter for both influx and efflux, leading to the excretion of NO_3_^−^ into the xylem. This essential function contributes to the movement of NO_3_^−^ from roots to shoots, highlighting the importance of these transporters in the nitrogen dynamics of plants [62,63]. AtSLAH1, a homolog of the Slow Anion Channel (SLAC), regulates long-distance Cl^−^ transportation from roots to shoots and participates in Cl^−^ loading of the xylem. The SLAC1 family represents a relatively recently discovered group of plant plasma membrane anion channels. The SLAC1 gene is highly conserved [64,65]. The Aluminum-Activated Malate Transporter (ALMT) protein family, which is exclusive to plants, acts as transporter for anion transfer across plasma membranes. When exposed to high external Cl^−^ conditions, certain ALMT members may facilitate substantial Cl^−^ efflux. This family, including AtALMT12 and AtALMT9, is crucial for Cl^−^ and nitrate transport regulation, particularly during stomatal closure and opening, respectively [66,67].

**Figure 3:**
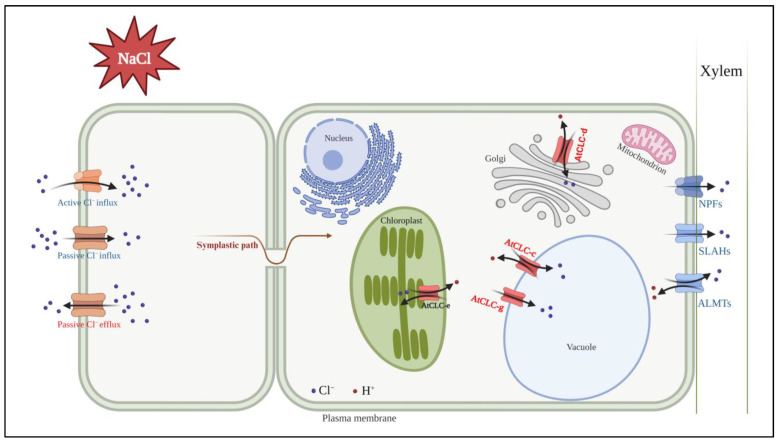
Model showing known Cl^−^ transporters affecting Cl^−^ transport and Cl^−^ tolerance under salt stress. Transporters upregulated in response to salt stress are indicated in red; those that are downregulated are indicated in blue. Among the seven AtCLCs (*Arabidopsis thaliana* CLC proteins), four are related to Cl^−^ transport. AtCLC-c and AtCLC-g are localized in the tonoplast. AtCLC-c and AtCLC-g have been suggested to play a role in Cl^−^ transport within the vacuole. AtCLC-d is localized in the Golgi apparatus, and AtCLC-d is known to regulate Golgi pH through Cl^−^ transport. AtCLC-e is found in the thylakoid membrane and has been reported to facilitate Cl^−^ transport, which is essential for efficient photosynthesis. NPFs, SLAHs, and ALMTs relate to Cl^−^ transport to xylem. The figure was generated using BioRender.com.

Among them, the predominant members of the ALMT, SLAC1, and NPF families serve primarily as plasma membrane anion channels. Although certain proteins within these families cooperate with CLC in Cl^−^ transport functions, their structural configuration is completely different from that of CLC proteins. In addition, the CLC family is predominantly localized to the endomembrane system of plant cells. They collectively establish a cell signaling network that facilitates the movement of Cl^−^. In Figure 3, an increase in Cl^−^ within plant organs in response to salt stress is shown. The upregulation of the CLC expression, including that of *AtCLC-c* and *AtCLC-g*, facilitated the influx of Cl^−^ into the vacuole. Simultaneously, the expressions of NPF, SLAH, and ALMT channels were downregulated, which suppressed the entry of Cl^−^ into the xylem. As a result, Cl^−^ was sequestered within the vacuole, effectively reducing the harmful effects of Cl^−^ stress in plants. The CLC protein family primarily distributes within the intracellular membrane system of plants. It is highly probable that CLC exchangers have a crucial function in intracellular fine-tuning processes, aiding the adaptation to Cl^−^ stress.

## 5. Discussion and Prospective Research

Salt stress causes an accumulation of Cl^−^ in plant cells, leading to toxicity. Understanding how plants transport Cl^−^ is crucial for plant salt tolerance. While our knowledge of Na^+^ transport mechanisms in relation to salt tolerance is more advanced, the transport of Cl^−^ is a complex process that involves chloride channels. Plant CLCs are important proteins found in the endomembrane system, and they play a significant role in plants’ response to salt stress. CLCs are also important for Cl^−^ transport as major Cl^−^ channels or exchangers in animals. The Cryo-EM structure of CLCs is relatively conserved, and this review compares these structures of CLCs in different species. CLCs primarily function as exchangers in plants. In the Cryo-EM structure of AtCLC-a, the Pro160 residue is crucial for nitrate delivery. Additionally, in plants, the size of the CLCs’ ion-conducting pore is associated with the preference for ion delivery. Therefore, our understanding of the transport mechanisms of other CLC transporters in plants is progressing.

The response of plants to salt stress and their tolerance levels vary among different species. It is important to compare and summarize how CLCs respond to salt stress in different plants. This review aims to systematically summarize the roles of known CLCs in land plants when it comes to salt stress. However, there is still much to learn about CLCs. This includes understanding how these proteins in the endomembrane system recognize, uptake, and transport Cl^−^ in different types of cells and under different conditions. Additionally, the regulatory mechanisms that govern these complex processes are still not fully understood.

In the future, it is crucial to thoroughly investigate the signaling network of CLC proteins and their involvement in long-distance transport of Cl^−^. Many important unknowns remain in this process, including the interaction between Cl⁻ and NO₃⁻ transporters, the maintenance of membrane potential, and the discovery of new transport pathways and regulatory factors. To unravel the genetic basis of plant salt tolerance, the use of genomic and machine learning approaches to fully exploit genetic diversity may be a promising research direction. In addition, the importance of changes in energy metabolism under saline–alkali conditions should be considered, particularly in studying how relevant proteins maintain a delicate balance between energy supply and consumption.

Furthermore, the potential of CLC family proteins in capturing fluoride ions (F^−^) warrants further investigation. Exploring their affinity for fluoride ions may contribute valuable insights into their broader functional roles and ecological relevance. Iodine ions (I^−^), F^−^, and Cl^−^ are all members of the halogen family. Seaweeds are known to accumulate noticeable amounts of iodine, and, given the role of CLC family proteins in ion transport, it is plausible to hypothesize their potential involvement in I^−^ uptake and accumulation.

## Figures and Tables

**Table 1:** CLCs in Arabidopsis thaliana.

Name	Location	Function	References
AtCLC-a	Tonoplast	NO_3_^–^ transport to vacuole, stomatal regulation	[28,38]
AtCLC-b	Tonoplast	NO_3_^−^ efflux from vacuole	[29]
AtCLC-c	Tonoplast	Cl^−^ transport to vacuole,stomatal regulation	[13,38]
AtCLC-d	Golgi	Cl^−^/NO_3_^–^ transport for pH regulation of trans-Golgi network, negative regulator of PAMP-triggered immunity (PTI)	[37,39]
AtCLC-e	Thylakoid	Cl^−^ homeostasis in thylakoid, photosynthetic electron transport	[35,36]
AtCLC-f	Golgi membrane	-	[36]
AtCLC-g	Tonoplast	Cl^−^ transport, phloem recirculation of chloride	[33]

**Table 2:** CLCs in different terrestrial plants.

Name	Species	Location	Function	References
CLC-Nt1	*N. tabacum* TN90	ER	Regulating the pH within the ER	[46]
CsCLC-c	*Poncirus trifoliata*	-	Cl^−^ accumulated in the roots and shoots	[17]
CsCLC-6/7	*Camellia sinensis*	-	Absorption and long-distance transport of Cl^−^	[47]
GhCLC-5/16	*Gossypium hirstum*	-	Transport, interaction and homeostasis of Cl^−^ and NO_3_^−^	[48,49]
GhCLCg-1	*Gossypium hirstum*	Tonoplast	Cl^−^ transport to vacuole	[50]
GmCLC-1	*Glycine max*	Tonoplast	Cl^−^ transport to vacuole	[43]
GsCLC-c2	*Glycine soja*	Tonoplast	Cl^−^ transport to vacuole	[44,51]
MhCLC-c1	*Malus hupehensis*	Plasma membrane	Cl^−^ transport	[52]
NtCLC2/13	*N. tabacum* K326	-	Cl^−^ transport	[53]
OsCLC-1/2	*Oryza sativa*	Tonoplast	Osmotic adjustment at high-salinity	[41,54]
OsCLC-6	*Oryza sativa*		Drought stress response	[55]
PgCLC-c1/c2/d	*Punica granatum*	-	Cl^−^ transport to vacuole	[56]
SaCLC-c1	*Suaeda altissima*	-	Cl^−^ transport	[57]
SaCLC-d/f/g	*Suaeda altissima*	-	Cl^−^ transport	[58]
TaCLC-a-6AS-1/c1-3AS/ e-3AL	*Triticum aestivum*	-	Cl^−^ transport	[59]
ZmCLC-d	*Zea mays*	-	Cl^−^ transport	[45]
